# Use of DNA barcode in the identification of fish eggs in tributaries of the Paranapanema River basin

**DOI:** 10.1590/1678-4685-GMB-2019-0352

**Published:** 2020-06-22

**Authors:** Moema Cristina Costa de Lima, Same Costa Lima, Camila Satie Savada, Karen Mayumi Suzuki, Mário Luís Orsi, Fernanda Simões de Almeida

**Affiliations:** 1 Universidade Estadual de Londrina, Departamento de Biologia Geral, Laboratório de Genética e Ecologia Animal, Londrina, PR, Brazil.; 2 Universidade Estadual de Londrina, Departamento de Biologia Animal e Vegetal, Laboratório de Ecologia de Peixes e Invasões Biológicas, Londrina, PR, Brazil.

**Keywords:** Reservoirs, conservation, freshwater, Neotropical, COI, ichthyoplankton

## Abstract

Fish eggs are often excluded from identification analysis since at this stage of development there are few morphological characters. The correct identification of eggs can provide important information about spawning areas of species. The current work aimed to identify fish eggs in the Tibagi and Cinzas Rivers using the DNA barcode to obtain information on richness and diversity, adding to the existing data in the area. Of the 928 sequences analyzed using the BOLD Systems database, 99.78% were able to be identified at a specific level, demonstrating a high success rate for egg identification. The samples resulted in 25 species, 11 families, and 2 orders. Of the 25 species found, more than half (60%) present reproductive migration behavior, indicating that the tributaries of the Capivara reservoir are being used as a migratory route by these species. Eggs of rare and endangered species were found, indicating these tributaries as spawning grounds for these species. The results demonstrate the importance of identifying fish eggs in reservoir-influenced environments to recognize breeding areas of native and endangered species, as well as the importance of the Tibagi and Cinzas Rivers for the maintenance of native fish species in the Paranapanema River.

## Introduction

Despite great diversity, freshwater ecosystems are one of the most threatened natural environments (Dudgeon *et al.*, 2006; Strayer and Dudgeon, 2010). Among the threats are destruction and degradation of habitat and the construction of reservoirs (Lévêque *et al.*, 2008; Geist, 2011). Dams are one of the main threats to biodiversity in freshwater environments due to the inability of some species to survive in dammed water bodies, as a result of loss of breeding habitats, impairment in reproductive migration, and population fragmentation. In addition, dams provide greater water transparency, allowing greater predation of fish eggs, larvae, and juveniles (Agostinho *et al.*, 2007).

The Paranapanema River, one of the main tributaries of the Paraná River (Orsi *et al.*, 2016), currently includes 11 hydroelectric plants along its main channel in a cascade reservoir system. The main tributaries of the Capivara reservoir are the Tibagi and Cinzas Rivers, considered important for maintaining the biodiversity of local ichthyofauna. As these rivers are lotic environments and similar to the original basin, they offer adequate conditions that aid in the maintenance of species (Dias *et al.*, 2004; Hoffmann *et al.*, 2005; Agostinho *et al.*, 2007; Lopes *et al.*, 2007; Vianna and Nogueira, 2008; Orsi, 2010).

In fact, identification, distribution, and abundance of ichthyoplankton are useful for identifying spawning places and migration routes in areas influenced by reservoirs (Nakatani *et al.*, 1997). However, the classification of these reproductive products is extremely complex, as many of their morphological characters are not yet sufficiently developed to be analyzed (Pegg *et al.*, 2006; Ward *et al.*, 2009). The task is even more problematic for eggs, due to their small size and similarity between different species. Therefore, identification using morphological characters does not include this stage, precisely because, in most cases, it is impossible to identify species (Richards, 2005).

The use of molecular markers to correctly discriminate species has become extremely important, among the molecular tools available, the technique proposed by Hebert *et al.* (2003) known as the DNA barcode method. One of the great advantages of this technique in resolving species from ichthyoplankton is that it can be applied to organisms at different stages of their life cycle, including eggs (Stoeckle, 2003). Some studies have already demonstrated the efficiency of the technique in identifying fish eggs and larvae (Hubert *et al.*, 2008; Gleason and Burton, 2012; García-Dávila *et al.*, 2014, 2015; Rodrigues *et al.*, 2018). Frantine-Silva *et al.* (2015) and Lima (2015) when identifying ichthyoplankton using the DNA barcode technique in Paranapanema river reservoirs, observed that the Capivara presented the highest species richness when compared to the other reservoirs, as well as its main tributaries, the Tibagi and Cinzas Rivers.

Due to the greater richness of species found in previous studies, the present study aims to identify fish eggs collected in three consecutive reproductive cycles (piracema periods 2012-2015) in the Tibagi River and Cinzas River using the DNA barcode technique, in order to obtain more information on the estimation of drifting eggs as well as the spawning grounds for fish species.

## Material and Methods

### Egg sampling and screening

**Figure 1:**
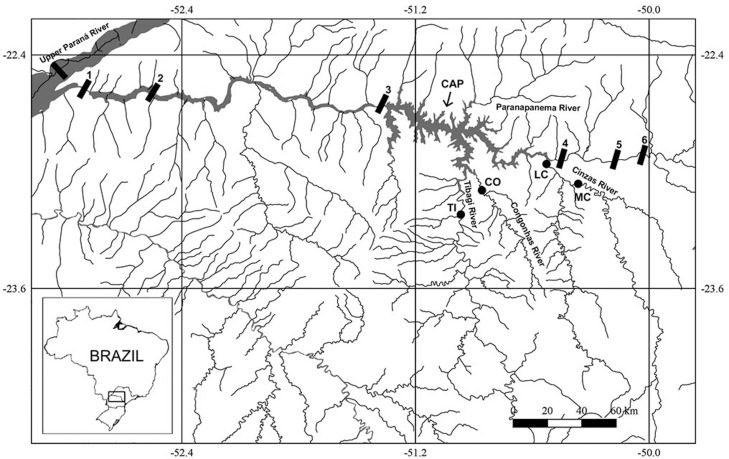
Distribution of sampling points along the lower-middle stretch of the Paranapanema River basin. CAP: Capivara reservoir, TI: Tibagi River, CO: Congonhas River, LC: lower Cinzas River, and MC: middle Cinzas River. The numbers represent the locations of the dams: (1) Rosana; (2) Taquaruçu; (3) Capivara; (4) Canoas I; (5) Canoas II; (6) Salto Grande.

Sampling was performed in two main tributaries of the Parapanema river, the Tibagi River and the Cinzas River, during three reproductive periods of fish (piracema) (October 2012 to March 2013, September 2013 to March 2014, and September 2014 to March 2015). In every piracema, monthly collections were carried out at each of the locations. The study points in the Tibagi river basin were represented by its sub-tributary, the Congonhas River, and the lower portion of its main channel, and the points in the Cinzas River basin were the middle and lower portions of its main channel (Figure 1).

For sampling, two conical nets with a 0.5 mm mesh, 1.6 m in length were used, remaining approximately 20 cm below the water surface. In each sampling, three repetitions of ten minutes were performed. The collected samples were immediately deposited in 98% alcohol and then stored at −20 °C. In the laboratory, the collected samples were screened using a stereoscopic magnifying glass. After pre-classification, samples were randomly selected to reach the greatest variety of egg morphotypes per sampling event, and subsequently photo-registered and individualized for further genetic analyses (Figure S1). The protocol was approved by the Ethics Committee of the State University of Londrina – CEUA/UEL (CEUA n° 29790.2012.39).

### DNA extraction, amplification, and sequencing of the 5' region of the COI gene

For total DNA extraction, each egg was placed in a 96 well plate (microtiter plates) and extraction was performed using Chelex100 solution (BIO-RAD) and Proteinase K (Invitrogen) according to the methodology described by Frantine-Silva *et al.* (2015). Amplification of the 5' region of the COI gene was carried out according to Frantine-Silva *et al.* (2015), using the primers FishF1 5'-TCAACCAACC ACAAAGACATTGGCAC-3' and FishR1 5'-TAGACTT CTGGGTGGCCAAAGAATCA-3' (Ward *et al.*, 2005). The purified samples were sequenced in 10 μL reactions containing 1 μL Big Dye buffer, 2 μL Big Dye Terminator v. 3.1. Cycle Sequencing (Applied Bio-systems, CA, USA), 0.25 μL of primer FishF1 (20 μM), and water to complete the volume. Reaction products were sequenced in an ABI-PRISM 3500 XL automatic sequencer (Applied Biosystems).

### Data analysis

The COI sequences were obtained only from the forward band, which was larger than 600 bp and the quality of the sequences was verified using the online application Electropherogram Quality Analysis (Togawa and Brígido, 2003), available at (http://asparagin.cenargen.embrapa.br/phph/). Next, alignment of the sequences was performed using the MUSCLE application (Edgar, 2004) in the MEGA program, v6.0 (Tamura *et al.*, 2013).

All sequences were submitted to the BOLD database (Ratnasingham and Hebert, 2007), (http://www.boldsystems.org/), to verify the correspondence and similarity of the submitted sequences with those stored in the database. The sequences deposited with the best matches for each taxon (> 99%), together with the sequences of the samples analyzed, were incorporated into the intraspecific and interspecific genetic distance analyses based on the Kimura-2-Parameters evolution model (K2P) (Kimura, 1980), which was chosen as it presents better performance when genetic distances are low (Nei and Kumar, 2000; Hebert *et al.*, 2003), as is the case of comparisons between species. Samples with low interspecific genetic divergence values (<2%) compared to sequences deposited with BOLD Systems were defined at species level and samples with intraspecific genetic divergence greater than (2%) were defined at genera and family level.

The same model was applied for the construction of a genetic distance tree using the Neighbor-Joining (NJ) method, aiming to obtain a graphical representation of the distribution of genetic distances between taxa. Distance analysis and identification of the second taxon with the shortest interspecific distance (nearest neighbor) as well as the Neighbor-Joining tree were performed using the MEGA v6.0 program (Tamura *et al.*, 2013). The sequences obtained were deposited in BOLD Systems, available at: BOLD projects: CAPV (Accession nos. CAPV001-17 to CAPV928-17).

## Results

### Molecular identification and genetic distance

A total of 928 sequences were obtained, with approximately 600 bp after the final alignment. Based on the similarity between the sample sequences and the sequences available in the database, it was possible to identify 926 of the 928 eggs analyzed (99.78%) at species level, with a mean similarity > 99%. Of the 928 eggs analyzed, 528 came from the Cinzas River tributary and 400 from the Tibagi River.

Analysis of the similarity of the sequences resulted in 2 orders, 11 families, 18 genera, and 25 species. Among the 928 samples, 274 were distributed in 16 species of the order Characiformes (64%) and 654 in 9 species of the order Siluriformes (36%). Only 2 specimens could be discriminated only at family level, with a mean similarity of (91.62%), both represented by the family Heptapteridae (Table S1).

All 25 species identified are present in the list of documented species for the Paranapanema River, thus representing 11.1% of a total of 225 species described for the Paranapanema River. The amplified fragments had a mean of 600 bp, with good quality, and no evidence of insertions, deletions, or stop codons. The distances based on K2P demonstrated that intraspecific distances were <1%, with a minimum distance of 0% and a maximum of 0.51%.

**Figure 2:**
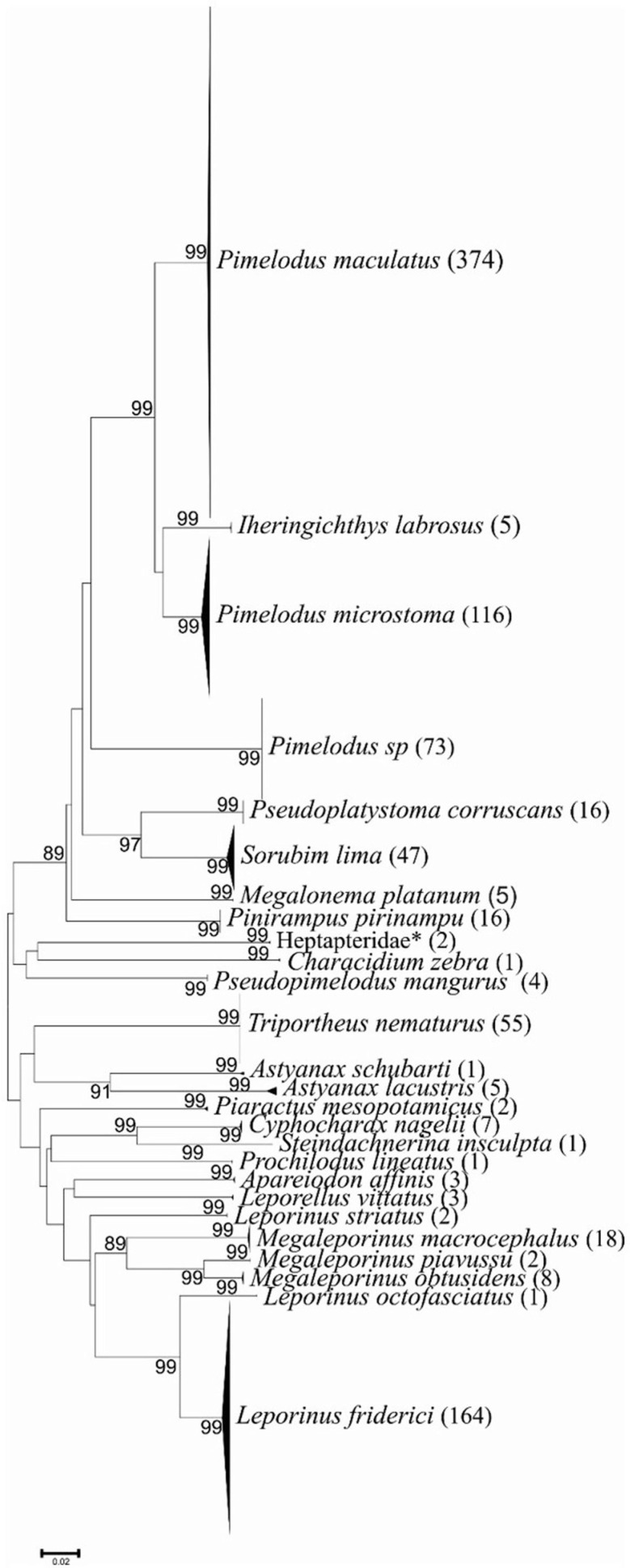
Dendrogram of genetic distances of species found. The Kimura-2-Parameters genetic distance related to the Neighbor-Joining method with 1000 pseudoreplicates using MEGA v6.0 software. Sustainability values of the branches are presented next to the nodes at each fork; values below 70% were hidden. The group names in italics indicate the lowest possible taxonomic level, and absolute abundance is indicated in parentheses. (*) Species for which sequences are not deposited in BOLD.

The compressed NJ-K2P tree points to a short distance between specimens identified at the species level and vouchers taken from the BOLD Systems (<1%), as well as high branch sustainability indices (Figure 2). Distance analysis to the nearest taxon (DVP) was performed to measure the distance between related taxa. The shortest DVP distance was observed between *Megaleporinus obtusidens* and *Megaleporinus piavussu* (D = 4.59 ± 0.90) (± 1).

### Taxonomic abundance and composition of sampled eggs

Regarding the two main tributaries studied, the lower Cinzas River presented the largest number of species (20), while the Tibagi River presented the lowest number (11) (Table 1). Among the 20 species found in the lower Cinzas River, *Pimelodus maculatus* (219), *Pimelodus microstoma* (74), *Leporinus friderici* (44), *Sorubim lima* (32), and *Megaleporinus macrocephalus* (18) were the most abundant. Of the 11 species found in the Tibagi River, *Pimelodus maculatus* (138), *Leporinus friderici* (98), *Pimelodus* sp (73), *Pimelodus microstoma* (38), and *Pinirampus pirinampu* (16) were the most abundant. The Congonhas River presented only one species *Triportheus nematurus* (23) (Table 1).

**Table 1:** Table of species distribution in the Capivara reservoir tributaries.

HPP Capivara						
**Species**	%	N	BC	MC	CO	TI
Characiformes						
**Anostomidae**						
*Leporellus vittatus* (Valenciennes, 1850)	0.32	3	2			1
*Leporinus friderici* (Bloch, 1794)	17.7	164	44	22		98
*Leporinus octofasciatus* Steindachner, 1915	0.1	1				1
*Leporinus striatus* Kner, 1858	0.22	2	1	1		
*Megaleporinus macrocephalus* Garavello & Britski, 1988†	1.94	18	18			
*Megaleporinus obtusidens* (Valenciennes, 1837)	0.86	8	8			
*Megaleporinus piavussu* Britski, Birindelii & Garavello, 2012	0.22	2	1	1		
**Characidae**						
*Astyanax lacustris* (Lütken, 1875)	0.53	5	5			
*Astyanax schubarti* Britski, 1964	0.1	1	1			
**Crenuchidae**						
*Characidium zebra* Eigenmann, 1909	0.1	1		1		
**Curimatidae**						
*Cyphocharax nagelii* (Steindachner, 1881)	0.75	7	7			
*Steindachnerina insculpita* (Fernández-Yépez, 1948)	0.1	1	1			
**Parodontidae**						
*Apareiodon affinis* (Steindachner, 1879)	0.32	3				3
**Prochilodontidae**						
*Prochilodus lineatus* (Valenciennes, 1837)	0.1	1	1			
**Serrasalmidae**						
*Piaractus mesopotamicus* (Holmberg, 1887)	0.22	2	1	1		
**Triportheidae**						
*Triportheus nematurus* (Kner, 1858)†	5.92	55	13	15	23	4
**Siluriformes**						
**Heptapteridae**						
Heptapteridae	0.22	2	1	1		
**Pimelodidae**						
*Iheringichthys labrosus* (Lütken, 1874)	0.53	5	1			4
*Megalonema platanum* (Günther, 1880)	0.1	1				1
*Pimelodus maculatus* Lacepède, 1803	40.3	374	219	17		138
*Pimelodus microstoma* Steindachner, 1877	12.5	116	74	4		38
*Pimelodus sp.*	7.87	73				73
*Pinirampus pirinampu* (Spix & Agassiz, 1829)	1.7	16				16
*Pseudoplatystoma corruscans* (Spix & Agassiz, 1829)	1.7	16	16			
*Sorubim lima* (Bloch & Schneider, 1801)†	5.06	47	32	15		
**Pseudopimelodidae**						
*Pseudopimelodus mangurus* (Valenciennes, 1835)‡	0.43	4	4			

**Subtitle:** (December 2012 to March 2015). (%): Relative frequency for each species (N): Absolute frequency, by collection point. LC: lower Cinzas; MC: middle Cinzas; CO: Congonhas River; and LT: lower Tibagi.

‡ Endangered species (Abilhoa & Duboc, 2004).

† Species non-native to the upper Paraná River (Júlio Jr *et al.*, 2009).

**Figure 3:**
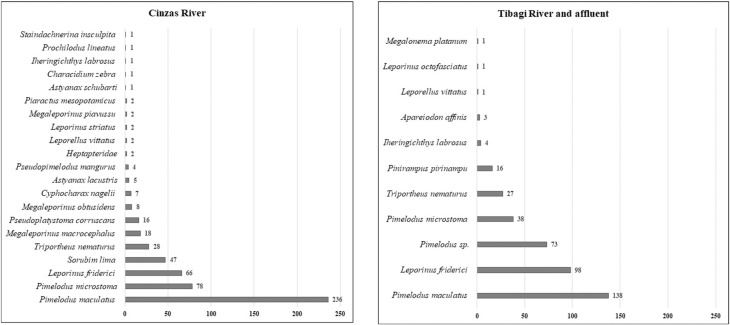
Species found for each tributary: Cinzas River and Tibagi River, numbers in the bars indicate the number of specimens.

When comparing the two tributaries studied, the highest richness was found for the Cinzas River, with 21 taxonomic units, which represented 84% of the species identified at specific level (Figure 3). Among the captured species, we observed the presence of *Pseudopimelodus mangurus*, a rarely captured species, categorized as vulnerable and the species *Pseudoplatystoma corruscans,* categorized as near threatened.

The Tibagi River and its sub-tributary (Congonhas River) presented a smaller diversity of species (11), representing 44% of the total identified. Eggs of *Leporellus vittatus*, *Iheringichthys labrosus*, *Triportheus nematurus*, *Pimelodus microstoma*, *Leporinus friderici,* and *Pimelodus maculatus* were found in both the Tibagi River and the Cinzas River. For both tributaries, the abundance and richness of native and migratory species overlapped with three non-native species identified; *Triportheus nematurus*, *Megaleporinus macrocephalus,* and *Sorubim lima;* the latter two found only in the Cinzas River. For the native species, the abundance of the short-distance migratory species *Pimelodus maculatus* stands out, totaling 374 of the total eggs identified (40.3%).

## Discussion

The differential of the present study is the analysis of fish eggs, where identification by morphological characters is not possible in most cases due to the size and interspecific similarities (Richards, 2005). By identifying almost 100% of the samples, this study is relevant, since fish egg identification analyses are scarce, particularly in the freshwater environment of the Neotropical region (Oliveira and Ferreira, 2008; Hermes-Silva *et al.*, 2009; Frantine-Silva *et al.*, 2015), since the majority of works are focused on the marine environment (Shao *et al.*, 2002; Burghart *et al.*, 2014; Harada *et al.*, 2015; Hubert *et al.*, 2015; Lin *et al.*, 2016; Hofmann *et al.*, 2017; Rodrigues *et al.*, 2018). Correct identification of fish eggs is crucial for recognizing and protecting breeding areas of commercially important species, as well as endangered species (Valdez-Moreno *et al.*, 2010).

Analysis of the sequences resulted in specific identification of 99.78% of the samples. This level of efficacy was also obtained in other studies using the same methodology for identifying reproductive fish products (García-Dávila *et al.*, 2014, 2015; Becker *et al.*, 2015; Frantine-Silva *et al.*, 2015; Hubert *et al.*, 2015; Rodigues *et al.*, 2018). The accuracy of species identification using the DNA barcode technique depends on the presence of high-quality reference sequences available in databases such as GenBank and BOLD Systems (Becker *et al.*, 2015). Thus, the optimal egg identification rate obtained at the species level is due to the fact that the upper Paraná River basin has a large barcode data set available for its ichthyofauna (Pereira *et al.*, 2013).

Two major studies carried out with ichthyoplankton in several reservoirs of the Paranapanema River demonstrated good identification efficiency using the DNA barcode method. Frantine-Silva *et al.* (2015) were able to identify 99.81% of a total of 536 samples and Lima (2015) was able to identify 88.44% of a total of 961 samples. The current study identified 928 eggs from the Cinzas and Tibagi Rivers, the main tributaries of the Capivara reservoir, during three piracema periods. In addition, the results contributed six new species not yet identified in previous studies by Lima (2015) and Frantine-Silva *et al.* (2015), such as: *Astyanax schubarti*, *Characidium zebra*, *Leporellus vittatus*, *Leporinus striatus*, *Pseudopimelodus mangurus,* and *Steindachnerina insculpta.* Nineteen of the 25 species identified in the current study were also found by Frantine-Silva *et al.* (2015) and Lima (2015) in the tributaries of the Capivara Reservoir, the Cinzas and Tibagi Rivers. Therefore, the total species identified by DNA barcode considering all analyzes in the Paranapanema River corresponds to approximately 29.8% of the species richness documented for this region (225) (Jarduli *et al.*, 2020).

Only two samples presented non-specific correspondence, with mean similarity of 91.62%, being classified only at the family level. This low similarity is probably associated with the great diversity of the Neotropical region, where it is common that some species have not yet have been adequately validated and deposited, as the region has very rich and diverse ichthyofauna, and although many species have already been described, many still await description (Levêque *et al.*, 2007; Albert and Reis, 2011; Toussaint *et al.*, 2016). Except for these two individuals, the other samples were able to be identified at a specific level with high reliability, including taxa with complex taxonomic identification, as in the case of the genus *Astyanax* and family Anostomidae, which can easily be misidentified using traditional taxonomic methods, even in adult individuals (Garavello and Britski, 2003; Bertaco and Garutti, 2007). Two species belonging to the genus *Astyanax: Astyanax lacustris* and *Astyanax schubarti* and seven species of the family Anostomidae: *Leporellus vittatus*, *Leporinus friderici*, *Leporinus octofasciatus*, *Leporinus striatus*, *Megaleporinus macrocephalus*, *Megaleporinus obtusidens,* and *Megaleporinus piavussu* were identified with a high match to specimens deposited in the database (> 99%) (Table S1).

Several factors influence the distribution patterns and structure of fish assemblages, as each species selects a spawning site based on a set of biotic and abiotic characteristics such as location, period, temperature, and duration and reproductive intensity of adults. Thus, to ensure a sufficient number of survivors, fish usually need adequate conditions to reproduce (Bialetzki *et al.*, 2005; Baumgartner *et al.*, 2008). However, environments influenced by dams lack the ideal conditions for the survival and reproduction of many South American fish species (Agostinho and Gomes, 2005; Agostinho *et al.*, 2008). Studies carried out on the Paranapanema River show that the tributaries present greater species richness than the reservoirs (Frantine–Silva *et al.*, 2015; Lima, 2015).

In the current study, reproductive products of 25 species were identified, among them 15 native species with reproductive displacement, which may carry out short migrations, such as: *Astyanax altiparanae* (Vazzoler, 1996), *Leporinus friderici*, *Apareiodon affinis*, *Iheringichthys labrosus*, *Steindachnerina insculpta* (Agostinho *et al.*, 2003), *Leporinus octofasciatus*, (Duke Energy, 2008) *Astyanax schubarti, Pimelodus maculatus,* and *Cyphocharax nagelii* (Suzuki *et al.*, 2004). Long-distance migrants were also identified: *Piaractus mesopotamicus*, *Prochilodus lineatus*, *Pseudoplatystoma corrusccans*, *Pinirampus pirinampu* (Agostinho *et al.*, 2003), and *Leporinus obtusidens* (Suzuki *et al.*, 2004). To complete their life cycles, long-distance migratory species require a habitat with the necessary conditions for spawning, development, and growth (Agostinho *et al.*, 2008; Pelicice *et al.*, 2015).

According to Shibatta *et al.* (2007), river fragmentation due to the construction of dams prevents many migratory and rheophilic fish species from completing the reproductive process. Analyzing fish eggs and larvae, Baumgartner *et al.* (2008) observed evidence of intense reproductive activities of migratory fish in tributaries of the Paraná River. The authors pointed out that the presence of tributaries free of dams plays a fundamental role in the maintenance of rheophilic species and provides conditions for the drift of eggs and larvae, making them important for maintaining regional ichthyofaunistic diversity.

Studying reproductive activities in the Capivara reservoir, Orsi (2010) found that among the points studied, reproductive activity was more intense in the stretches of the Tibagi and Cinzas Rivers, with semilotic and lotic characteristics respectively. The rheophilic and migratory species are highlighted, indicating a strong influence of tributaries in the reproduction of these fish species. This intense reproductive activity must also be associated with the environmental characteristics of these tributaries, such as the presence of macrophyte banks, submerged trunks, and forest remnants, as well as the occurrence of marginal lagoons. In the present study, more than half of the species identified (60%) have a habit of reproductive displacement, indicating that the tributaries of this reservoir are being used as a spawning site by these species, making the preservation of these areas essential for the maintenance of the local ichthyofauna. The highest abundance was observed for *Pimelodus maculatus* (representing 40.30% of the captures), which may be related to its better adjustment to reservoir conditions than other species (Frantine-Silva *et al.*, 2015).

Among the 1,497 samples of reproductive products (eggs and larvae) processed in Paranapanema river reservoirs by Lima (2015) and Frantine-Silva *et al.* (2015), the presence of *Pseudopimelodus mangurus*, a rare and endangered species, had not been observed. In total, four *P. mangurus* eggs were identified in our analyses, all from the lower Cinzas River locality. Frantine-Silva *et al.* (2015) found another endangered species in the same location, *Steindachneridion scriptum* (Abilhoa and Duboc, 2004), and appointed this region as a critical point for species conservation. For the Cinzas River, it is also worth mentioning the presence of a large number of fish eggs of commercial importance, found in low frequency in the Paranapanema River, such as the *Piaractus mesopotamicus*, popularly known as Pacu, and *Pseudoplatystoma corruscans* known as Pintado (Kubitza, 1998; Hilsdorf and Marques, 2006; Duke Energy, 2008). The latter is found in the Red Book of Threatened Fauna of Paraná in the category of near threatened (Abilhoa and Duboc, 2004). The presence of eggs of this species indicates that these fish are using the tributary during their reproductive cycle. The Cinzas River presented higher species richness than the Tibagi River, which was also observed in the work of Frantine-Silva *et al.* (2015). According to Hoffmann *et al.* (2005), the Cinzas River is the environment farthest from the lacustrine zone of the reservoir, being less influenced by the dam, and presenting characteristics more similar to the original environment. Thus, this stretch is of prime importance for the Capivara reservoir in terms of species maintenance.

The Tibagi River is the largest tributary of the reservoir and second largest stretch with respect to diversity and has been pointed out by several studies as responsible for maintaining diversity (Shao *et al.*, 2002; Hoffmann *et al.*, 2005). This river presented species of short and long reproductive displacement, indicating it as an alternative route to complete reproductive cycles. Among these species, eggs of *Pinirampus pirinampu* were found, a large species with long reproductive displacement which, according to Dias *et al.* (2004) uses the Capivara reservoir as a place of growth and food but chooses the lotic environments of its main tributaries as a breeding area. In addition, the presence of *Megalonema platanum* was also observed, a rare species in the Paraná River basin and a species for which little information is reported about its biology. For the Congonhas River, a tributary of the Tibagi River, only one species was found, *Triportheus nematurus*. However, this result is probably due to the small number of sampled eggs collected at this point, since this tributary still maintains adequate conditions that favor the conservation of fish including native and migratory species (Garcia *et al.*, 2019).

Eggs from species not native to the basin were found in both tributaries, although a relatively small number of samples and species. *Triportheus nematurus* was present in both tributaries while *Sorubim lima* and *Megaleporinus macrocephalus* were found only in the Cinzas River. These three species are alien species and, according to Júnior *et al.* (2009), originate from invasion after the construction of the Itaipu reservoir. Although *Megaleporinus macrocephalus* and *Triportheus nematurus* dispersed due to the elimination of the Seven Falls barrier, the removal of obstacles was not the only reason for their introduction. *Megaleporinus macrocephalus* was heavily restocked (Agostinho *et al.*, 2007), with leaks recorded from aquaculture tanks during flooding (Orsi and Agostinho, 1999). Similarly, the species *Triportheus nematurus* was intensively introduced for restocking purposes in several reservoirs located in the upper Paraná River basin (CESP, 1996).

In conclusion, the results obtained demonstrated that the DNA barcode was fundamental for the identification of species of fish egg samples, aggregating information regarding the reproductive biology of fish species present in two important tributaries of a basin impacted by cascading reservoirs. Therefore, we recommend using this technique in studies of fish eggs for future conservation plans.

## Supplementary Material

The following online material is available for this article:

Figure S1 - Egg morphotypes defined a priori.

Table S1 Species identified from eggs from the main tributaries of the Capivara reservoir.
