# Supplementary material for: Use of DNA barcode in the identification of fish eggs in tributaries of the Paranapanema River basin
Source: Genet Mol Biol. 2020 Jun 22;43(3):e20190352. doi: 10.1590/1678-4685-GMB-2019-0352 (PMC7315765; doi:10.1590/1678-4685-GMB-2019-0352)
Supplement: Supplementary file 1 [file 1415-4757-GMB-43-3-e20190352-s1.pdf]

Genetics and Molecular Biology
https://doi.org/10.1590/1678-4685-GMB-2019-0266

Supplementary Material to "Use of DNA barcode in the identification of fish eggs in tributaries of the Paranapanema River basin"

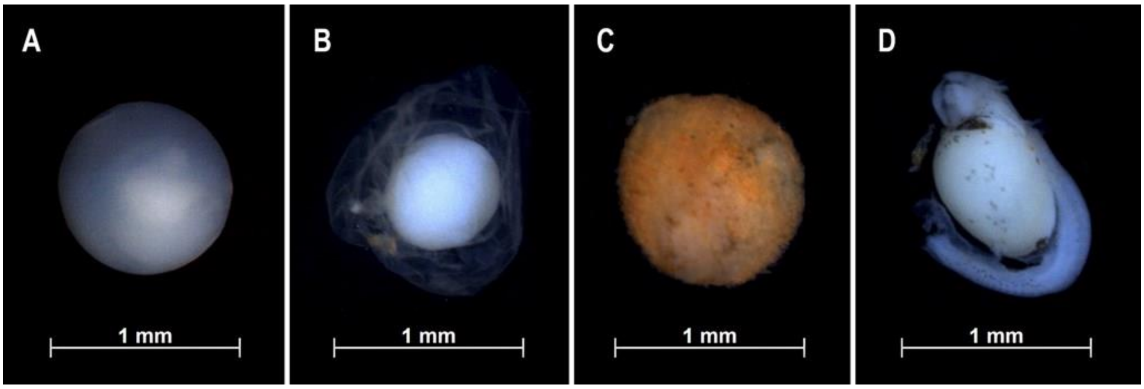

Figure S1 - Egg morphotypes defined a priori. A: egg with reduced perivitelline space (ID: 14J; *Pimelodus sp*.), B: developed perivitelline space (ID: 1405Ac; *Steindachnerina insculpta*), C: egg with adhered organic matter (ID: 1411Ac; *Pimelodus maculatus*), and D: hatched egg (ID: 1427Ac; *Pimelodus microstoma*).
