# Supplementary material for: Use of DNA barcode in the identification of fish eggs in tributaries of the Paranapanema River basin
Source: Genet Mol Biol. 2020 Jun 22;43(3):e20190352. doi: 10.1590/1678-4685-GMB-2019-0352 (PMC7315765; doi:10.1590/1678-4685-GMB-2019-0352)
Supplement: Supplementary file 2 [file 1415-4757-GMB-43-3-e20190352-s2.pdf]

Genetics and Molecular Biology
https://doi.org/10.1590/1678-4685-GMB-2019-0266

Supplementary Material to "Use of DNA barcode in the identification of fish eggs in tributaries of the Paranapanema River basin"

Table S1 - Species identified from eggs from the main tributaries of the Capivara reservoir.

Species	N	BOLD-SE	D ± SE	NN	DNN ± SE
**CHARACIFORMES**					
**Anostomidae**					
Leporellus vittatus (Valenciennes, 1850)	**3**	99,95 ± 0,06	0,08 ± 0,09	*Apareiodon affinis*	17,27 ± 1,85
Leporinus friderici (Bloch, 1794)	**164**	99,97 ± 0,01	0,10 ± 0,05	*Leporinus octofasciatus*	6,69 ± 1,11
Leporinus octofasciatus Steindachner, 1915	**1**	100 ± 0	0,00 ± 0,00	*Leporinus friderici*	6,69 ± 1,11
Leporinus striatus Kner, 1858	**2**	100 ± 0	0,00 ± 0,00	*Leporinus friderici*	14,86 ± 1,65
Megaleporinus macrocephalus Garavello & Britski, 1988	**18**	99,92 ± 0,02	0,12 ± 0,09	*Megaleporinus piavussu*	12,91 ± 1,70
Megaleporinus obtusidens (Valenciennes, 1837)	**8**	99,98 ± 0,02	0,04 ± 0,04	*Megaleporinus piavussu*	4,59 ± 0,87
Megaleporinus piavussu Britski, Birindelii & Garavello, 2012	**2**	100 ± 0	0,00 ± 0,00	*Megaleporinus obtusidens*	4,59 ± 0.87
**Characidae**					
Astyanax lacustris (Lütken, 1875)	**5**	99,84 ± 0,11	0,39 ± 0,15	*Astyanax schubarti*	16,39 ± 2,02
Astyanax schubarti Britski, 1964	**1**	99,50 ± 0	0,51 ± 0,28	*Astyanax lacustris*	16,39 ± 2,02
**Crenuchidae**					
Characidium zebra Eigenmann, 1909	**1**	99,82 ± 0	0,18 ± 0,18	*Leporellus vittatus*	21,20 ± 2,14
**Curimatidae**					
Cyphocharax nagelii (Steindachner, 1881)	**7**	99,97 ± 0,03	0,04 ± 0,04	*Steindachnerina insculpita*	12,93 ± 1,69
Steindachnerina insculpita (Fernández-Yépez, 1948)	**1**	100 ± 0	0,00 ± 0,00	*Cyphocharax nagelii*	12,93 ± 1,69
**Parodontidae**					
Apareiodon affinis (Steindachner, 1879)	**3**	99,94 ±0,06	0,18 ± 0,12	*Leporellus vittatus*	17,27 ± 1,85
**Prochilodontidae**					
Prochilodus lineatus (Valenciennes, 1837)	**1**	100 ± 0	0,00 ± 0,00	*Leporinus octofasciatus*	17,05 ± 1,85
**Serrasalmidae**					
Piaractus mesopotamicus (Holmberg, 1887)	**2**	100 ± 0	0,34 ± 0,19	*Megaleporinus piavussu*	17,81 ± 1,87
**Triportheidae**					
Triportheus nematurus (Kner, 1858)	**55**	100 ± 0	0,00 ± 0,00	*Prochilodus lineatus*	20,58 ± 2,07
**SILURIFORMES**					
**Heptapteridae****	**2**	91,62 ± 0,09	0,17 ± 0,16	*Pseudopimelodus mangurus*	21,52 ± 2,13
**Pimelodidae**					
Iheringichthys labrosus (Lütken, 1874)	**5**	99,93 ± 0,04	0,09 ± 0,09	*Pimelodus microstoma*	6,25 ± 1,02

Species	N	BOLD-SE	D ± SE	NN	DNN ± SE
Megalonema platanum (Günther, 1880)	**1**	100 ± 0	0,00 ± 0,00	*Pimelodus microstoma*	15,91 ± 1,78
Pimelodus maculatus Lacepède, 1803	**374**	99,99 ± 0	0,16 ± 0,09	*Pimelodus microstoma*	6,24 ± 1,08
Pimelodus microstoma Steindachner, 1877	**116**	99,97 ± 0,01	0,21 ± 0,07	*Pimelodus maculatus*	6,24 ± 1,08
Pimelodus sp	**73**	99,99 ± 0	0,02 ± 0,01	*Pimelodus maculatus*	15,7 ± 1,74
Pinirampus pirinampu (Spix & Agassiz, 1829)	**16**	100 ± 0	0,00 ± 0,00	*Iheringichthys labrosus*	16,18 ± 1,73
Pseudoplatystoma corruscans (Spix & Agassiz, 1829)	**16**	100 ± 0	0,00 ± 0,00	*Sorubim lima*	10,80 ± 1,33
Sorubim lima (Bloch & Schneider, 1801)	**47**	99,96 ± 0,01	0,16 ± 0,10	*Pseudoplatystoma corruscans*	10,80 ± 1,33
**Pseudopimelodidae**					
Pseudopimelodus mangurus (Valenciennes, 1835)	**4**	100 ± 0	0,00 ± 0,00	*Pimelodus sp*	19,75 ± 2,00

Subtitle: N: Number of captures; BOLD (%): Mean similarities to the nearest taxon found through the Boldsystems Identification System ®; D (%): Mean genetic distance within the groups; NN (%): Nearest Neighbor; DNN (%): Distance to nearest taxon; SE (%): Standard error. Similarity indices considered in this study: <2% specific correspondence; > 2% congeneric or family level.
